# The Human Brain: Its Structure, Physiology and Diseases: With a Description of the Typical Forms of Brain in the Animal Kingdom

**Published:** 1848-10

**Authors:** 


ART. V.—*The Human Brain: its Structure, Physiology and Diseases: with a description of the typical forms of brain in the animal kingdom.* By SAMUEL SOLLY, F. R. S., senior assistant surgeon to St. Thomas hospital, etc., etc. From the second London edition, with one hundred and eighteen wood engravings. Philadelphia. Lea & Blanchard, 1848. 8 vo. pp. 496.

The first English edition of this work, was published in 1836. The present American republication, is of the second edition, which has been very recently issued in London, and has not only been revised by the author, but large additions have been made, especially to the departments of physiology, and diseases of the brain.

One half of the volume is devoted to the structure of the brain. The author's investigations in this department, have been conducted upon the plan enunciated in the following quotation from the preface:—

"The only philosophical method of simplifying and giving a character of general interest to the anatomy of the human brain, is by commencing with the structure and functions of the nervous system in the lowest and simplest forms of animal existence, rising by degrees to the highest, carefully observing each addition of parts, and the relationship borne by these to an addition of function. By pursuing this course, we shall be rewarded by finding that the encephalon, this apparently most complicated organ in the human being, is but a gradual development from an extremely simple fundamental type on one uniform and harmonious plan, and that the seeming complexity of the cerebro-spinal axis in man, really arises from the great concentration, as opposed to the extreme diffusion, of its component parts in the lower order of animals; for in no particular are the higher orders more strikingly distinguished from the lower than in the concentration of function within circumscribed spaces."

Every philosophical student must appreciate the great advantage which has been derived from adopting a method of examining and describing the brain, different from that heretofore pursued, and still followed in most systematic works on anatomy. The latter consists in dissecting from the superior surface of the hemispheres, downward, by removing the cerebral substance in successive slices. This, as the author remarks, is rather a destructive, than a dissecting process, and has doubtless retarded, instead of promoted knowledge of the structure and functions of this portion of the organism. For conclusive evidence of the superiority of commencing dissections of the base, (the merit of which, Mr. Solly attributes to Gall and Spurzheim,) and proceeding by unfolding parts, so as to discover their natural connections and relations, the reader has only to consult this work. He will also be equally convinced of the great advantage and interest which the study of the human brain has derived from comparative anatomy, by tracing the successive developements of the nervous system, from the simplest function in the bodies of animals, through the several gradations in the scale of organization, up to the summit, as exemplified in man.

Mr. Solly proposes, also, some changes in nomenclature, which, to say the least, serve to diminish, in no small degree, the complexity of the pursuit in this particular. Without professing to have given this portion of the work that close attention which its merits claim, and which we intend to bestow upon it hereafter, we have no hesitation in saying, that it is the most complete, and scientific treatise with which we are acquainted, and, as such, we commend it most cordially to those who wish to become conversant with all that is now known, or may be rationally conjectured respecting this most interesting and important portion of the human fabric.

The part devoted to the physiology of the brain, occupies but a few pages. The author, however, very judiciously, in the anatomical division of the subject, connects with the description of parts, his views of their respective functions. The physiological chapter, therefore, contains only a *resume* of physiological facts, previously presented in that connection. The following quotations embody the author's opinions respecting the functions of the more important components of the cerebral structure:—

"In the course of our observations of the composition and properties of neurine, and on the essential elements of a nervous system, the following fundamental principles have been established, and need only be adverted to in the present section.

1. That vesicular neurine is the source of power.
2. That medullary neurine is the conductor of it.
3. That medullary neurine is also the conductor of those impressions which call forth the power of the vesicular neurine.
4. That the vesicular neurine is collected in masses of variable form and size—the *ganglia*.
5. That the medullary neurine is moulded into cords and bands—the *nerves* and *commissures*.

"The *spinal cord* is a series of ganglionic centres, structurally homologous and functionally analogous to the jointed ganglionic cord of the articulata, and although we are unable to point out any corresponding anatomical lines of demarkation between them, they are as functionally distinct as the auditory, optic, and olfactory ganglia of the brain.

"The *medulla oblongata* consists of three ganglia on each side of the mesial line—six, therefore, in all. The olivary bodies, most probably the lingual ganglia, the restiform or pneumogastric ganglia, the posterior pyramidal bodies or auditory ganglia. The olivary ganglia are connected with the rest of the cerebral ganglia by means of the olivary commissures, and the important office of those ganglia, if my hypothesis is correct, that they preside over the consensual movements of the tongue, as an organ of speech, explains the reason of such a perfect communication with the rest of the encephalon. Between the pneumogastric ganglia and the brain the commissural communication is not so distinct, and there does not appear to be the same physiological reason to expect it.

The auditory ganglia are imbedded in the sensory tract; but undoubtedly some of these fibres which we have heretofore considered as belonging solely to this system of nerves, must be regarded as belonging to that system of longitudinal commissures which we have seen so distinctly carried out in the brain."

Of the *Cerebellum* he says:—

"There can, I think, be little doubt but it is a regulator and co-ordinator of muscular action on the one part, most probably by means of the central portion of the cerebellum, viz., the superior and inferior vermiform processes. On the second part, it certainly would appear to hold some relation to the generative function. The pathological and other facts adduced by Drs. Gall, Vimont, and Broussais, on this subject, are very striking, and almost as conclusive as all other physiological evidence.

The *locus niger* in the crus cerebri is the next ganglion for our consideration: it is the serial homologue and analogue of the anterior peaks of gray matter of the spinal cord. It is, I suppose, the seat of the excito-motor power of the third pair of nerves, the importance of which in relation to the instinctive and conservative movements of the eyeball need not be insisted on here.

The *tubercula quadrigemina* or optic tubercles, we may fairly conclude, are the instruments by which the physical impressions of light received by the retina are converted into sensations of light, color, form, &c.

The optic thalami and corpora striata, or anterior and posterior cerebral ganglia, are the next in rotation. With regard to the office of these nervous centres, we have already had occasion to consider the thalamus as the essential ganglion of the sensory tract, as the corpus striatum is of that of the motor tract. And I am quite disposed to adopt the ingenious and philosophical theory of my friend, Dr. Carpenter, as enunciated in his review of Mr. Noble's work in the October number of the British and Foreign Quarterly Medical Review for 1846.

The anterior and posterior cerebral ganglia are regarded by Dr. C. as forming part of the series of sensorial centres, of which we have seen other members in the olfactory, optic, and auditory ganglia. That they are independent centres of action, not mere appendages to the hemispheric ganglia, appears from the large quantity of vesicular neurine which they contain; and that the corpora striata are so, further appears from the absence of any correspondence in size between them and the hemispheric ganglia. Thus in fishes, we find that the corpora striata make up the principal bulk of the second pair of masses; in reptiles, birds, and the lower Mammalia, they still form a very large portion of that which is commonly termed the cerebrum; and their subordinate aspect in man and the higher Mammalia is solely due to the large relative developement of the hemispheric ganglia. On the other hand, there is scarcely any rudiment of the thalami optici to be discovered in fishes; their proportional size increases in reptiles, birds, and the lower mammalia; but it is only in man that their dimensions approach those of the corpora striata. The peculiar connection of the thalami optici with the posterior columns of the spinal cord, and their great development in man, suggest the idea that they are the ganglia of *tactual* sensation; whilst the connection of the corpora striata with the anterior column indicates their relation with the motor function. The very close relation between the thalami optici and the corpora striata—corresponding, as Messrs. Todd and Bowman have suggested, with that which exists between the posterior and anterior peaks of gray matter in the spinal cord—harmonizes well with the fact that the greater number of muscular movements are directed by common sensation; whilst the special connection established by the inter-cerebral commissure between the corpora striata and the optic ganglia (tubercula quadrigemina) explains the peculiar influence of the sense of light in directing ceriain classes of muscular actions. The communication which is formed by the medullary substance of the cerebrum between these ganglia and the hemispheric ganglia seems to be the medium by which *sensations* are transmitted to the latter, to become the stimulus of intellectual operations, and by which the influence of *volition* is transmitted downwards to excite muscular motions through the corpora striata.

The whole chain of sensory ganglia is regarded by Dr. C. as not only the instrument by which sensations are received, but also as the centre of those *automatic* muscular movements which differ from those of a simple reflex character, in being dependent upon sensation. To this head, he refers the purely *instinctive* actions of the lower animals, as well as a variety of actions performed by the human being, both in health and disease; such as the consensual movements of the eyes, the regulation of the laryngeal muscles in the production of vocal sounds, the convulsive movements in hydrophobia, brought on by the sight or sound of water, &c. &c. And he considers the actions which become automatic by *habit*, as executed through the same channel; each movement being directly prompted by the sensation with which it has become assocaated.

We come lastly to those important ganglia which crown and cover in the rest—the hemispherical. If there is one point in the physiology of the brain more unequivocally demonstrated than another, it is that these ganglia are the instruments of the mind—the portion of the brain in which sensations are converted into perceptions, and give rise to ideas. Comparative anatomy: developmental anatomy: experiments on living animals; observations on its size and form, as indicated by the size and form of the skull; and last, but not least, pathology,—all afford a mass of overwhelming evidence that this portion of the brain, and this only, is the cerebral organ of intellectual power."

Mr. Solly is of opinion, that there is much truth in phrenology. He discusses the correctness of this opinion at some length. The following quotation, contains a summary of the reasons which lead him to think favorably of that science.—

My reasons for believing that there must be a great deal of truth in phrenology are fourfold. First, I have received from practical phrenologists, and especially the late worthy Mr. Deville, such accurate characters of individuals known to me, but unknown to them, that I cannot believe the accounts I received could be the result of accident and conjecture, which must have been the case if phrenology is untrue.

Secondly. Phrenology alone—as it appears to me—can account for all the varieties of insanity, especially monomania.

Thirdly. The facts which have been collected by the late Mr. Deville. showing that the brain will alter its form at any period of life.

Fourthly. The existence of longitudinal commissures.

*The diseases of the brain* occupy more than two hundred pages of the work. These are arranged under the following heads:—1. anæmic affections, 2. hyperæmic, 3. convulsive, 4. organic.

The author adopts the views of Dr. Burrows, with respect to the circulation within the cranium, and gives extended extracts from Dr. Burrow's work, noticed in the last number of this Journal.

*Delirium Tremens,* he classes among the anæmic affections of brain. As some qualification of this opinion, it should be stated, that he applies the term delirium tremens exclusively to cases in which delirium supervenes upon sudden withdrawal of alcoholic stimulus. Those cases in which delirium occurs during the use of stimulants, and as a direct consequence of their use, he distinguishes as *delirium ebriosorum.* The latter he considers quite a different affection, involving irritation and congestion of the brain. We quote a paragraph in which the circumstances marking the distinction are mentioned:—

It is impossible to give in words, all that distinguishes these two diseases; they must be seen frequently to be appreciated. But the following will assist in their diagnosis. The head and skin generally is cool and moist in delirium tremens, dry and hot in delirium ebriosorum. The pupil varies in both according to the stage: in the early stage of both it is generally contracted, in the latter stage dilated. The conjunctiva injected and red in delirium ebriosorum; the reverse in delirium tremens. The mental derangment in the former is more allied to an exalted, excited state of intellect; in the latter it approaches fatuity and depression. The tongue is generally pale and furred in delirium tremens, sometimes unnaturally clean and red; in delirium ebriosorum is usually dry, and sometimes brown, but this is no certain guide. The pulse is most uncertain, for as all inflammatory affections of the brain are depressing in their effects on the heart's action, so do we find that the pulse is not hard and wiry in the hyperæmic affection, which, however, never amounts to one acutely inflammatory. Still, on the whole, there is less power in the beat of an artery, and that more varied in delirium trements than in delirium ebriosorum."

Mr. S. treats cases of delirium tremens on the stimulant plan. Delirium ebriosorum, of course, according to the author's pathology, is aggravated by stimulants, and requires depletion, carefully practiced.

The subject of *Anæmic Coma* is next treated of. We regard this as one of the most important subjects in practical medicine. The coma occurring in the course of diseases productive of exhaustion, particularly in children, is still, we fear, treated, by many practitioners, as an affection requiring depletory measures. We commend the facts and principles presented under this head to the careful consideration of the reader. Our limits will not permit us to present an analysis of them, nor to notice the remainder of the work, except to enumerate [the subjects of which the author treats. After anaemic coma follows ramollisement. He then proceeds to speak of hyperaemic affections. Under this head he considers inflammation of the hemispherical ganglion, in other words, the cineritious or vesicular element of the brain's structure, and inflammation of the neninges. *Apoplexy* falls within this division, which is considered at considerable length. *Convulsive Affections* follow next in order, and epilepsy. Lastly *organic diseases of the brain.*

The portion of the work devoted to diseases of the brain contains much that the practitioner will find both interesting and profitable. After what we have said respecting the portion of the work treating of anatomical details, it will, perhaps, be proper to add, in this connection, that the foregoing opinion is based upon a careful perusal of the portion occupied with diseases.

In conclusion, we feel confident that we do not overrate the merits of this book when we pronounce it one of the best of the many which have lately emanated from the medical press.

